# A cluster of X-linked miRNAs are de-repressed with age in mouse liver and target growth hormone signaling

**DOI:** 10.3389/fragi.2023.1261121

**Published:** 2023-10-10

**Authors:** Anna P. Petrashen, Yufei Lin, Bianca Kun, Jill A. Kreiling

**Affiliations:** Department of Molecular Biology, Cell Biology and Biochemistry, Center for the Biology of Aging, Brown University, Providence, RI, United States

**Keywords:** aging, miRNA, mir-465, growth hormone receptor, growth hormone signaling, liver

## Abstract

Growth hormone (GH) signaling influences lifespan in a wide variety of mammalian species. We previously reported that a cluster of miRNAs located on the X-chromosome are de-repressed with age in male mouse liver, and a subset, the mir-465 family, can directly attenuate expression of the growth hormone receptor (GHR) *in vitro* leading to a reduction in GH signaling. Here we show that this cluster of miRNAs is also upregulated in the liver with age in females, and that calorie restriction and the Ames dwarf genotype, both known to delay aging, attenuate the upregulation of the miRNA cluster. Upregulation of mir-465 *in vivo* leads to a reduction in GHR mRNA in the liver and an attenuation of GH signaling, indicated by a reduction in GHR, IGF-1, IGFBP3, and ALS mRNA expression. There is a corresponding reduction in IGF-1 protein levels in the liver and plasma. These results suggest that the age-associated upregulation of the X-chromosomal cluster of miRNAs could influence lifespan.

## Introduction

Studies have shown that alterations in insulin and insulin-like growth factor 1 (IGF-1) signaling can affect healthspan and lifespan in a large number of organisms ranging from worms to humans (Min and Tatar, 2018; Aguiar-Oliveira and Bartke, 2019; Bartke, 2021; Bartke, 2022). The lifespan extending effects in mammals are believed to occur through the actions of growth hormone (GH) signaling through the somatotropic axis. GH is synthesized in the anterior pituitary gland and is secreted into the bloodstream. Once in the circulation GH can bind to the GH receptor (GHR) in the liver. Activation of the GHR results in the phosphorylation of Janus-family tyrosine kinase 2 (JAK2), which subsequently phosphorylates the STAT5b transcription factor, stimulating IGF-1 expression (Alvarez-Nava and Lanes, 2017). Many studies to date examined the effects of a global reduction in GH signaling by knocking out components of the GH signaling pathway in model systems. The majority of these studies revealed an extension in healthspan and lifespan (Brown-Borg, 2015). These results are strengthened by methylome studies showing a deceleration of the epigenetic clock in Ames dwarf mice, a long-lived mutant strain with decreased GH signaling (Wang et al., 2017)**.** These results may apply to humans as a subset of centenarians had IGF-1 receptor (IGF1R) gene polymorphisms that may affect IGF1R activity (Suh et al., 2008). The Itabaianinha cohort, consisting of individuals who have a mutation in the GH releasing hormone receptor, and individuals with Laron dwarfism, which results from a mutation in the GHR gene, have an increased healthspan, however they do not have an extended lifespan (Aguiar-Oliveira et al., 2010; Guevara-Aguirre et al., 2011). Taken together these results suggest that a life-long reduction in GH signaling has a beneficial effect on healthspan and lifespan.

The timing of altered GH signaling appears to be important in determining the effects on healthspan and lifespan. GH signaling declines with age, and this decline is a risk factor for developing several age-associated conditions including chronic liver disease, diabetes, cardiovascular disease, sarcopenia, osteoporosis, and frailty (Rincon et al., 2004; Barzilai et al., 2012; Bartke et al., 2016). GH-deficient mice that receive GH during the juvenile period do not have extended lifespans, whereas administering GH to GH-deficient mice during adulthood does not affect the lifespan extension seen in these mice (Panici et al., 2010). An induced global reduction in GHR following the juvenile period showed differential effects in males and females, but overall was beneficial to both sexes, especially in female mice that showed a lifespan extension (Junnila et al., 2016; Duran-Ortiz et al., 2021). In contrast, tissue-specific reductions in GH signaling showed more negative effects. A lifelong reduction in the GHR in the liver led to conditions that are consistent with decreased healthspan and lifespan (Fan et al., 2009; List et al., 2014). A liver-specific reduction in IGF-1 in middle aged mice resulted in liver inflammation, accelerated bone loss, oxidative stress in different tissues and increased hepatic tumors (Gong et al., 2014). These data suggest that a global reduction in GH signaling results in increased lifespan, whereas a reduction in GH signaling only in the liver may reduce healthspan and ultimately lifespan.

GH production declines naturally with age and is linked to the development of age-related conditions. The mechanisms behind the age-associated reduction in GH signaling are poorly understood. We recently showed that a cluster of microRNAs (miRNAs) located on the X-chromosome that are expressed in testes and pluripotent stem cells are de-repressed with age in mouse liver. A subset of these miRNAs, the mir-465 cluster of miRNAs, are predicted to target nine mRNAs in the PI3K-AKT pathway that are reduced with age in RNA-seq analyses, including the GHR, indicating that these miRNAs may directly influence signaling pathways known to be associated with healthspan and lifespan. We subsequently showed that the mir-465 family directly targets the GHR mRNA transcript resulting in attenuation of GH signaling in a cultured mouse liver cell line (Elias et al., 2019). Here we expand on our previous studies to show that despite being located on the X-chromosome the upregulation of this cluster of miRNAs occurs equally in males and females. We also show that the upregulation of the cluster is attenuated by interventions and genetic backgrounds known to extend lifespan. Importantly, we show that members of the mir-465 family act to reduce GH signaling in the liver *in vivo*. These results suggest that the age-associated increase in mir-465 may contribute to the decline in GH signaling with age and may have implications on healthspan and lifespan.

## Materials and methods

### Mouse tissue

Male and female mice of strain C57BL/6N were obtained from the National Institute on Aging (NIA) Aged Rodent Colonies (www.nia.nih.gov/research/dab/aged-rodent-colonies-handbook). Liver tissue from male Ames dwarf, a long-lived strain containing a mutation in the *Prop1* gene resulting in growth hormone deficiency, and wild type control mice; and male calorie restricted and *ad lib* fed control C57BL/6 mice were obtained from the NIA Aged Rodent tissue bank (https://www.nia.nih.gov/research/dab/aged-rodent-tissue-bank-handbook). All procedures were approved by the Brown University IACUC and carried out in compliance with IACUC guidelines. This study was carried out in compliance with the ARRIVE guidelines.

### miRNA mimics

MicroRNA mimics for mmu-mir-465b-5p were purchased from Exiqon (Woburn, MA, United States). Negative Control 5 miRCURY LNA miRNA Mimic (GAU​GCU​ACG​GUC​AAU​GUC​UAA​G) was used as a control (Exiqon, Woburn, MA, United States).

### Transfection reagents

Primary hepatocytes were transfected with miRNA mimics using Fugene HD transfection reagent (Promega, Madison, WI, United States) at a 4:1 ratio of the transfection reagent to µg of nucleic acid following the manufacturer’s instructions. miRNA mimics were prepared for injection into mice by complexing 2 pmoles of mimic with Invivofectamine 3.0 (ThermoFisher, United States) following the manufacturer’s recommendations.

### miRNA injections

Mice were gently warmed under a heat lamp, placed in a restraining device, and the tail warmed by wrapping it in a paper towel wetted with warm tap water. The tail was dried and wiped with 70% ethanol. The tail vein was visualized by illuminating the underside of the tail using Veinlite R (Veinlite). Each mouse received a 200 µL injection of containing 2 pmoles of the miRNA mimic complexed with Invivofectamine 3.0.

### RNA isolation

RNA was isolated using a combination of Trizol reagent and the RNeasy kit (Qiagen, Valencia, CA, United States). 30–50 mg of tissue was homogenized in 1 mL Trizol and extracted with 200 µL of chloroform. Following centrifugation at 12,000 x g for 15 min the RNA in the aqueous phase was precipitated by adding 500 µL isopropanol. The precipitated RNA in solution was loaded onto the RNeasy column and purified according to the manufacturer’s instructions. The RNA was quantified using a NanoDrop spectrophotometer.

### Nanostring analysis of miRNA expression

Total RNA was isolated as above and diluted to a concentration of 33.3 ng/μL. 3 μL of each sample was run on the Nanostring platform using the nCounter Mouse v1.5 miRNA panel according to manufacturer’s instructions. Data was normalized to the top 100 expressed miRNAs on the panel using the nCounter software (Nanostring).

### 5′ and 3’ rapid amplification of cDNA ends (RACE)

The full-length transcripts encoding the miRNA cluster on the X chromosome were obtained using the SMARTer RACE 5’/3’ Kit following the instructions provided by the kit (Takara Bio United States Inc., San Jose, CA, United States). Briefly, total RNA was converted to cDNA using the kit reagents and instructions. The cDNA was used as a template for PCR reactions using gene specific primers (Table 1) and primers to the adaptors provided in the kit and SeqAmp polymerase (Takara Bio United States, Inc.). The PCR products were gel purified and either cloned into pRACE provided with the kit and sent for Sanger sequencing, or the purified PCR product was sent to the MGH CCIB DNA Core for complete amplicon sequencing. For the latter, reads were aligned to the mm10 genome using RNA star and assembled using Cufflinks.

**TABLE 1:** Primer sequences used for cloning, sequencing, and verifying the lncRNA encoding the mir-465 family.

Primer name	Sequence
465lncF1	TGC​CAT​TGT​AAT​AAC​ATG​CAG​GC
465lncR1	CAA​GAC​AAT​ACG​GGG​GAC​TGT
465lncE4F5	GTC​CCC​CGT​ATT​GTC​TTG​GT
465lncE4R5	TGC​AAA​TGT​AGC​TTC​CAT​GAT​CC
465lncF10	GCA​AAC​CCT​TTT​GTA​GCT​TCT​GT
465lncR10	CAT​AGT​CAC​ACA​GAG​GAG​CCA​G
465lncF11	TTG​GTA​CTC​AGT​AGT​TTG​ACA​GCA
465lncR11	GGC​AGA​GTT​CTC​AGA​CAT​GGT
465lncF13	TCT​GCA​GCA​TTC​TTC​TGG​CTC
465lncR13	GCA​AAT​GTA​GCT​TCC​ATG​ATC​CTC
465lncF15	GGC​TCC​TCT​GTG​TGA​CTA​TGG
465lncR15	TGC​CTT​CAA​TTT​GCC​AAA​CCA​A
465ra5LR1^a^	GAT​TAC​GCC​AAG​CTT​TCC​ATA​GTC​ACA​CAG​AGG​AGC​CAG​AAG​AAT​GCT​G
465ra5LR2^a^	GAT​TAC​GCC​AAG​CTT​TCA​CAC​AGA​GGA​GCC​AGA​AGA​ATG​CTG​CAG​AGA
465ra5MR1^a^	GAT​TAC​GCC​AAG​CTT​TCA​TGG​TGG​CAG​AGT​TCT​CAG​ACA​TGG​TCC​T
465lnc1.2bF	CAT​AAG​GAA​TTG​AAG​TGT​ACT​GCC
465lnc1.2bR	CCA​AGA​CAA​TAC​GGG​GGA​CT
465lnc1.3aF	CAT​AAG​GAA​TTG​AAG​TGT​ACT​GCC
465lnc1.3aR	AAG​GCA​CAG​AAC​GTT​AGC​TCT

^a^ These primers were used with the SMARTer RACE Kit (Takara Bio United States) and contain a sequence for In-Fusion cloning at the 5′ end.

### Quantitative real-time PCR

For quantification of mRNA expression 1 μg of total RNA was transcribed into cDNA in 50 μL reactions using random hexamers and the TaqMan kit (Applied Biosystems, Foster City, CA, United States), according to the manufacturer’s protocol. A volume of 0.1–1.5 μL of this reaction was used in subsequent qPCR reactions. For quantification of miRNA expression cDNA was made following the procedure outlined in Balcells et al. (2011). A quantity of 1 µg of total RNA was polyadenylated at the 3′ end by incubating for 30 min at 37°C with 0.25 U/µL polyA polymerase (New England Biolabs, Ipswich, MA, United States), 1 mM ATP (New England Biolabs, Ipswich, MA, United States), 5.5 mM MgCl_2_ in 1x reverse transcription (RT) buffer from the TaqMan kit (Applied Biosystems, Foster City, CA, United States) in a 25 µL volume. A degenerate RT-primer was added to a final concentration of 200 nM and allowed to anneal at 60°C for 5 min. Finally, the RNA was reverse transcribed to cDNA by incubating for 1 h at 42°C with 500 µM (each) dNTP mix (Applied Biosystems, Foster City, CA, United States), 1.25 U/µL MultiScribe reverse transcriptase (Applied Biosystems, Foster City, CA, United States), 5.5 mM MgCl_2_ in 1x RT buffer in a final volume of 50 µL.

qPCR was performed using the SYBR Green system (Applied Biosystems, Foster City, CA, United States) on an ABI ViiA 7 Real Time System instrument (Applied Biosystems, Foster City, CA, United States), according to the manufacturer’s specifications. Primers for mRNAs were designed using the Primer-BLAST software. Primers for miRNA quantification were designed using miRprimer (Busk, 2014). Primer sequences can be found in Table 2 **.** All primers were tested on serial dilutions of target cDNA to ensure they amplified quantitatively, i.e., had a slope between −3.0 and −3.6 and an R-squared value above 0.95 (Dorak, 2006). Only primers meeting these criteria were used in this study. Due to the short length of the miRNAs, we were not able to identify suitable primers for all of the miRNAs present in the X-chromosomal cluster. The forward and reverse primers were used at a concentration of 300 nM each. GAPDH and HPRT were used as normalization controls for mRNA expression. The small nucleolar RNAs SNORD66 and SNORD70 were used as normalization controls for miRNA expression. Significance was determined using 2-tailed Student’s t-test.

**TABLE 2:** qPCR Primer Sequences.

Target	Forward primer	Reverse primer
GAPDH	AGG​TTG​TCT​CCT​GCG​ACT​TC	TGT​CAT​ACC​AGG​AAA​TGA​GCT​TG
mir-465b	CGC​AGT​ATT​TAG​AAT​GGT​GCT​GA	GGT​CCA​GTT​TTT​TTT​TTT​TTT​TCA​GA
SNORD66	CGT​GTC​TGG​GCC​ACT​GAG​A	CAG​TTT​TTT​TTT​TTT​TTT​CCT​CAG​GT
SNORD70	TGG​AAC​TGA​ATC​TAA​GTG​ATT​TAA​CAA​A	CCA​GTT​TTT​TTT​TTT​TTT​TCT​CAG​TG
GHR	GTG​CAA​CCT​GAT​CCA​CCC​AT	CTC​CAC​GAA​TCC​CGG​TCA​AA
IGF-1	CAG​GCT​CAG​AGC​ATA​CCT​GC	AGC​AGG​TCA​GAG​TGG​GTA​CT
IGFBP3	AAC​CTG​CTC​CAG​GAA​ACA​TCA	AAC​TTG​GAA​TCG​GTC​ACT​CGG
IGFALS	GGA​ACA​ATG​GCT​CTG​AGA​ACA​G	GAT​GCT​CCA​GGA​TCT​GTC​CC
HPRT	TCC​CAG​CGT​CGT​GAT​TAG​CGA​TG	GGC​CAC​AAT​GTG​ATG​GCC​TCC​C

### Biotinylated miRNA mimic pulldown

Four-month-old male and female mice were injected with 2 nmoles of biotinylated mir-465b via the tail vein as described above. After 4 days the mice were sacrificed and the liver and blood were collected. 100 mg of frozen liver was homogenized on dry ice, thawed in PBS + 1.5% paraformaldehyde, and incubated for 20 min. The fixation was quenched with glycine for 5 min and the tissue washed with PBS + 60 U/mL of the RNase inhibitor Superase (ThermoFisher) + cOmplete protease inhibitor cocktail (Roche) added at the manufacturer’s recommended concentration and centrifuged for 5 min at 500 x g. The pellet was resuspended in lysis buffer (20 mM Tris + 100 mM KCl + 5 mM MgCl_2_ + 0.3% v/v NP40) + 60 U/mL Superase + 1x protease inhibitor cocktail. The suspension was incubated on ice for 20 min, while pipetting up and down 20x every 5 min with a p1000 pipet. Debris was removed by centrifugation at 10,000 x g for 30 min. 35 µl of the supernatant was removed as input. The remaining supernatant was incubated with streptavidin coated magnetic beads at 4°C for 4 h with rotation. Beads were subsequently washed 5x with 1 mL lysis buffer then incubated for 20 min at 37°C with 100 µL DNase (Qiagen). Beads were washed once with 500 µL of lysis buffer, then incubated in 100 µL lysis buffer + proteinase K (New England Biolabs) at 55°C for 20 min. One hundred µl of lysis buffer + proteinase K was added to the input and incubated at 55°C for 20 min. 750 µl of Trizol LS was added to the beads and input, and RNA was isolated following the manufacturer’s recommendations, except the samples were incubated at −20°C overnight after isopropanol addition, and 2 µL of GlycoBlue (ThermoFisher) was added to each sample to visualize the RNA pellet. mir-465b enrichment was determined by qPCR as described above using primers specific for mir-465b.

### Hepatocyte isolation

Hepatocytes were isolated following the protocol outlined by Zhang (http://www.mouselivercells.com/Documents/Hepatocyte%20Isolation%20Protocol.pdf). Briefly, the liver was perfused with ∼70 mL HBSS + 0.5 mM EGTA (without magnesium and calcium) through the portal vein to remove the blood, followed by ∼70 mL digestion medium (DMEM-low glucose w/L-Gln and Na-pyruvate + 1x Penn-Strep + 15 mM HEPES containing 100 CDU/mL collagenase). Once digestion was complete, the liver was excised and placed in a 10 cm dish containing ∼ 20 mL of the digestion medium. The liver was torn apart to release the hepatocytes and cell clumps were disrupted by trituration with a 25 mL pipet. The cell suspension was filtered with a 70 µm filter (Fisher Scientific). Cells were collected by centrifugation at 50 × g for 2 min at 4°C and washed 3x in 25 mL isolation medium (DMEM-high glucose w/L-Gln and Na-pyruvate + 1 mM Na-lactate + 2 mM L-glutamine + 15 mM HEPES + 1x Penn/Strep + 0.1 µM Dexamethasone +10% FBS), collecting the cells by centrifugation at 50 × g for 2 min at 4°C between washes. The final pellet was resuspended in 25 mL cold isolation medium. Viable cells were counted on a hemocytometer and the percent viability was determined by staining 10 µL of the cell suspension with 10 µL 0.4% trypan blue. Cells were diluted to 3 × 10^5^ viable cells/mL and cells were distributed by plating 10 mL of cells in 10 cm collagen-coated dishes. Once the cells attached to the plates, the medium was changed to culture medium (DMEM-low glucose w/L-Gln and Na-pyruvate + 10 mM Na-lactate + 2 mM L-glutamine + 5 mM HEPES + 1x Penn/Strep + 0.01 Dexamethasone).

### IGF-1 ELISA

Heparin stabilized plasma was collected from the mice at the time of sacrifice. Liver protein extracts were prepared by homogenizing 5–10 mg of tissue in 60 μL/mg tissue of tissue extraction buffer containing 100 mM Tris (pH 7.4) + 150 mM NaCl + 1 mM EGTA + 1 mM EDTA + 1% v/v Triton X-100 + 0.5% sodium deoxycholate + 1 mM phenylmethylsulfonyl fluoride + 1X cOmplete mini protease inhibitor cocktail. Samples were homogenized using either the Fisher PowerGen 125 motorized homogenizer, or by passage through a 26 G needle. Homogenized extracts were incubated on ice for 20 min, then centrifuged at maximum speed for 10 min at 4°C. The resulting supernatant was diluted 1:20 with MilliQ water prior to assessment of concentration using the Qubit Protein Assay kit (Qiagen, Q33211).

Liver and plasma total IGF-1 protein abundance was quantified using the IGF-1 (Mouse/Rat) ELISA kit (ALPCO) as per manufacturer’s protocol. Liver extracts and plasma samples were diluted 1:1 and 1:100, respectively, in assay buffer. All samples were run in duplicate. Absorbance readings were normalized to standard curve generated from readings of standard solutions of known IGF-1 concentration. IGF-1 measurements for liver extracts were normalized to protein concentration as determined by the Qubit Protein Assay kit.

### Statistical analyses

**FIGURE 1:**
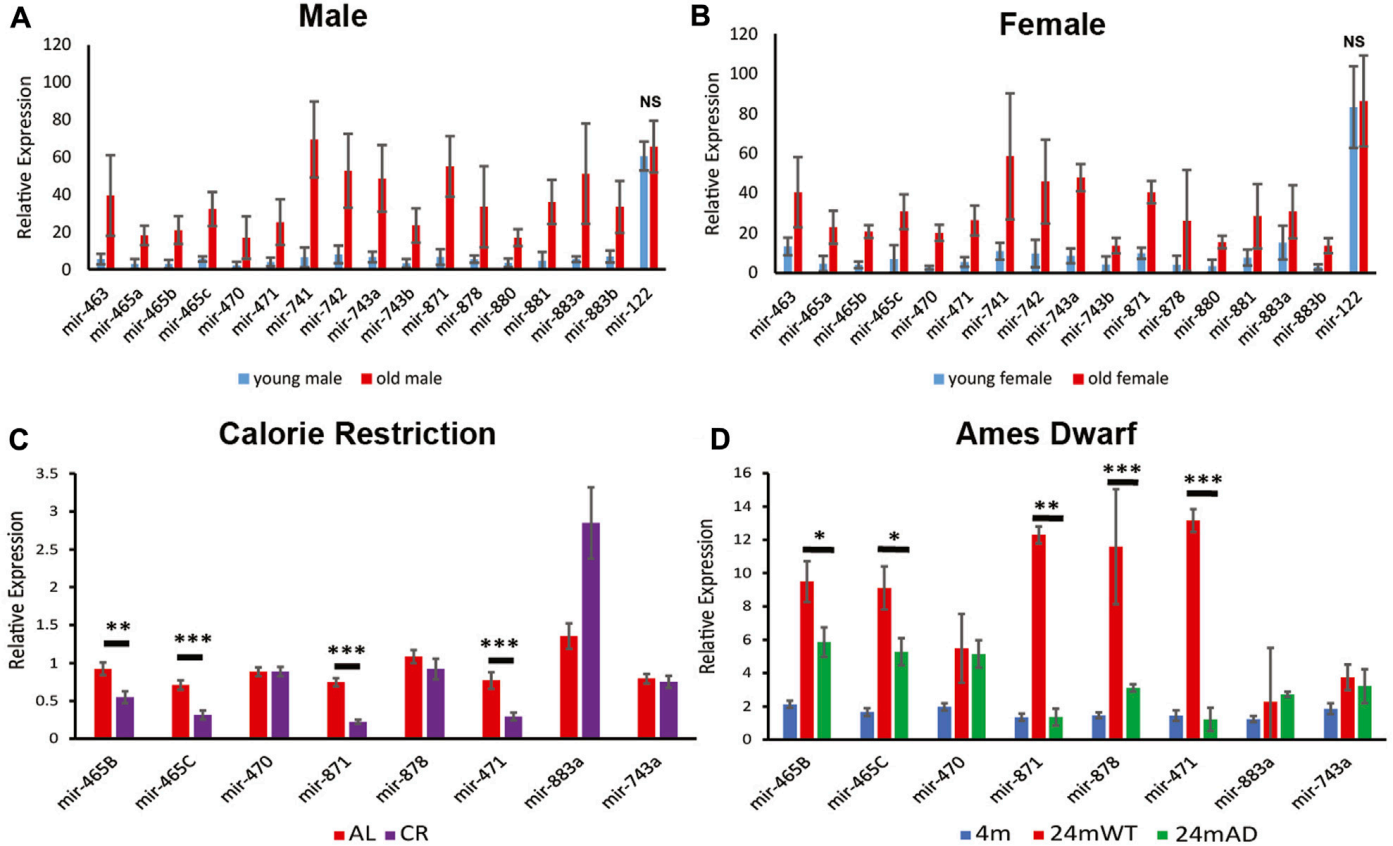
Expression of the X-chromosomal miRNA cluster. Relative expression levels for young (3 months) and old (24 months) male **(A)** and Female **(B)** mouse liver determined by Nanostring analysis. Note, all of the miRNAs in this cluster increase in expression by 24 months in wild-type mice. *p* < 0.001 and 0.02 for the miRNAs in the cluster in males and females, respectively. Normalized expression of the stably expressed liver-specific mir-122 is shown as a control to document that there is not a general increase in expression of all miRNAs in the aged mice. NS, not significant; *p* = 0.49 and 0.82 for miR-122 in males and females, respectively. Error bars represent standard deviation. **(C)** Quantitative PCR (qPCR) reveals that calorie restriction attenuates the increase in expression in male mouse liver for several of the miRNAs at 24 months of age as determined by qPCR. **(D)** The long-lived Ames dwarf genetic background attenuates the increase in miRNA expression for several of the miRNAs in male mouse liver, determined by qPCR. Error bars represent standard error of the mean (S.E.M.). AL, *ad libitum* fed, CR, calorie restriction. *n* = 6–9. ***, *p* < 0.001; **, *p* < 0.01; *, *p* < 0.05.

Statistical significance of the miRNA expression data was determined using the nSolver 4.0 software (Nanostring). Statistical significance of qPCR data was determined using a Student’s t-test for data with a normal distribution and a Mann-Whitney test for data without a normal distribution using either GraphPad Prism version 9.4.1 or Microsoft Excel 2019. Error bars in Figures 1A, B are standard deviation. The error bars on all remaining graphs are standard error of the mean (S.E.M.).

## Results

### Expression of the X-chromosomal miRNA cluster increases with age in males and female mice

Our previous studies showed an age-associated increase in expression of a cluster of 18 miRNAs located in a ∼65 kb region between the *Fmr1* and *Slitrk2* genes on the X-chromosome in male mouse liver (Elias et al., 2019). The updated mm10 genome annotation reveals there are 19 miRNAs in this cluster. To determine if this upregulation occurs in both sexes, or is restricted to males, we performed Nanostring analysis on young (3 months) and aged (24 months) male and female mice. Analysis of the expression of approximately 600 miRNAs confirmed that the X-chromosomal cluster of miRNAs that are present on the Nanostring card were upregulated at 24 months of age in both male and female liver (Figures 1A, B). Eighteen of the 19 miRNAs are represented on the Nanostring card, with the newly annotated 19th miRNA being absent. Since mir-465b and mir-465c are duplicated in the cluster, and the duplicates contain the identical sequence, it is impossible to distinguish mir-465b-1 from mir-465b-2 and mir-465c-1 from mir-465c-2. These miRNAs are represented as mir-465b and mir-465c on the card, respectively. These results indicate that despite being expressed in a testes-specific manner in males during development (Zhang et al., 2019a), the age-associated upregulation of the miRNAs in this cluster was a generalized phenomenon that occurs in both males and females. The level of increase was similar in each sex, indicating that the inactivated X chromosome was likely not involved in the upregulation seen in females and that similar age-associated mechanisms occurred in both sexes. In addition, we did not see the same increase in mir-122, a liver-specific miRNA (Figures 1A, B), showing that the increase in expression seen for the miRNAs in the X-chromosomal cluster were specific for that cluster and not a generalized phenomenon.

### Increase in expression is attenuated by calorie restriction and in Ames dwarf mice

To confirm that the upregulation of this cluster of miRNAs at 24 months resulted from physiological changes that occurred with age, we examined an intervention known to delay the development of aging phenotypes to determine if it attenuated or prevented the age-associated increase in expression of the X-chromosomal miRNA cluster. We examined expression of these miRNAs in liver from 24-month-old mice subjected to calorie restriction. We found reduced expression in liver from calorie restricted mice for the miRNAs located at the 5′ end of the cluster (based on the direction of transcription, as the miRNAs in this cluster are transcribed from the reverse strand) (Figure 1C). There was significantly lower expression of mir-465b and mir-465c in the calorie restricted animals. There was also reduced expression of mir-871 and mir-471, both located in the center of the cluster. Interestingly, calorie restriction led to an increase in mir-883a, suggesting that expression of this miRNA was induced by calorie restriction. There was no attenuation of expression levels of mir-470, mir-878, or mir-743a, the latter located at the 3′ end of the cluster. These results indicated that calorie restriction attenuated the rise in expression of a subset of miRNAs located towards the 5′ end of the X-chromosomal miRNA cluster, and that there may be differential regulation of expression of the miRNAs along the cluster, especially towards the 3’ end.

We investigated whether genetic backgrounds that are associated with an extended lifespan also show attenuation of the de-repression of the miRNA cluster with age. The Ames dwarf mouse is homozygous for a single mutation in the Prop-1 locus resulting in disruption of the somatotropic axis leading to an 50%–64% increase in lifespan (Sornson et al., 1996). When compared to 4-month-old animals on the same genetic background, wild type littermates had a robust increase in expression of the miRNA cluster at 24 months of age, whereas the Ames dwarf littermates showed attenuation of this increase (Figure 1D). These results indicated that lifespan extending genetic alterations may attenuate or delay the onset of upregulation of this cluster of miRNAs.

### The mir-465 family is processed from introns located in a long non-coding RNA that is increased in expression with age

**FIGURE 2:**
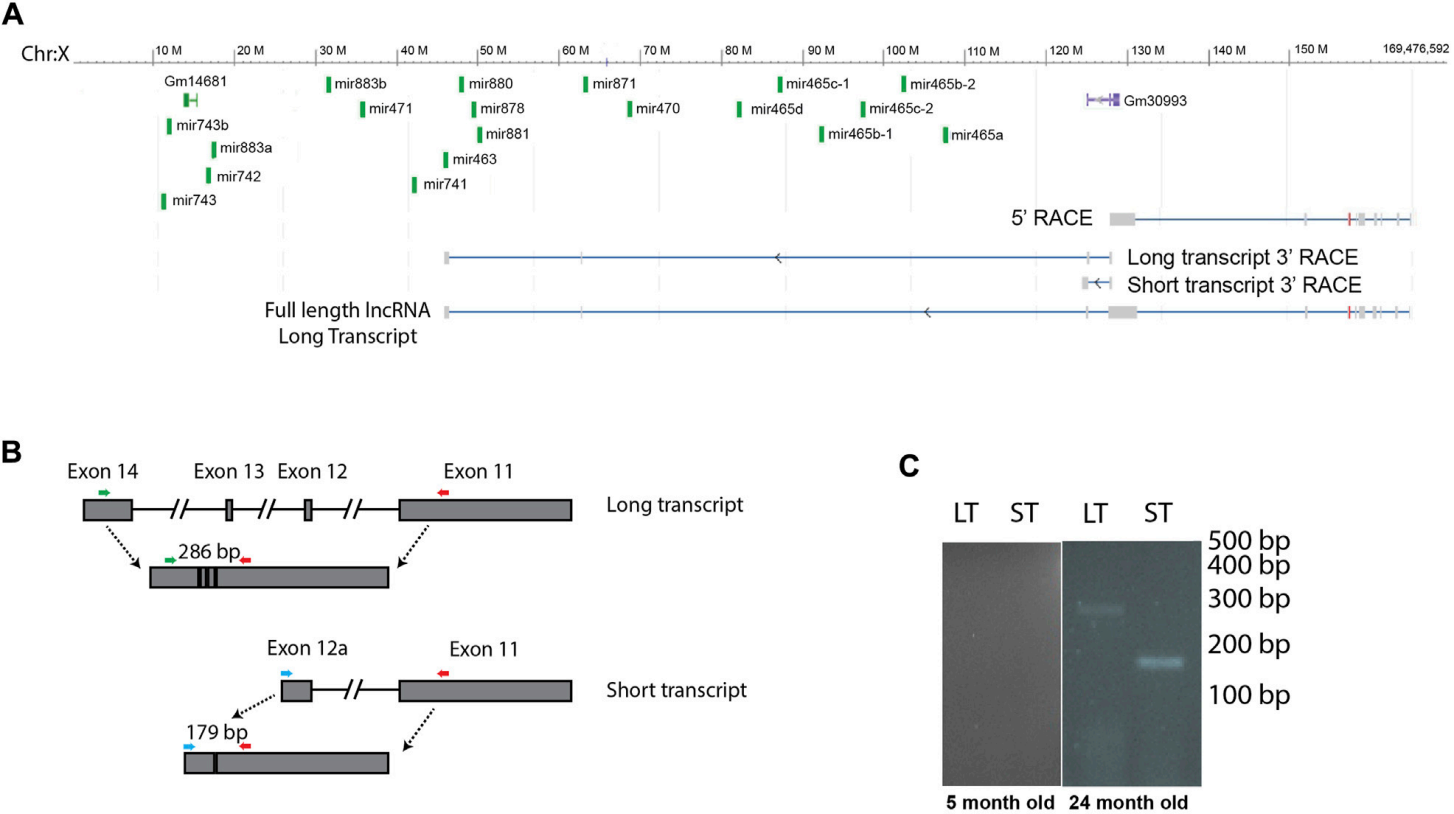
Several of the X-chromosomal cluster of miRNAs are encoded by a lncRNA. **(A)** We identified 2 transcripts by 3′ RACE that are encoded in the region of the miRNA cluster, a long transcript that contains 12 of the miRNAs located in introns, and a short transcript that stops before the miRNA genes. The full-length long transcript, identified by 5′ and 3′ RACE, is indicated at the bottom of the image. Note, this lncRNA is transcribed from the minus strand. **(B)** We designed primers to distinguish between the long and short transcript, colored arrows. Note primers are not drawn to scale. **(C)** RT PCR shows that both transcripts are expressed in old animals, and neither is expressed in young animals. This figure shows cropped lanes from different gels run on different days. LT, long transcript, ST, short transcript.

Many miRNA clusters are transcribed as a polycistronic transcript. To investigate whether this cluster of miRNAs is expressed as a single transcript, or if the miRNAs are expressed on multiple transcripts and therefore may be subject to different regulatory processes, we investigated transcripts originating from this region. We identified a long, non-coding RNA (lncRNA) that was transcribed from the negative strand that could function as a primary miRNA. This lncRNA encoded 12 miRNAs found in the X-chromosomal cluster, including the mir-465 family. This primary miRNA overlapped with a previously published primary miRNA sequence found in the Neuro2A cell line (Chang et al., 2015), however the transcript that was expressed in liver was longer. Using the Neuro2A primary miRNA sequence as a starting point, we performed 5′ and 3′ RACE to identify the 5′ and 3′ ends of the transcript in mouse liver. We found that the transcription start site of the primary miRNA is approximately 62 kb upstream of the transcription start site of the first miRNA in the cluster, mir-465a (Figure 2A). We used whole amplicon sequencing to determine the 3’ end of the transcript. We found 2 transcripts that are expressed from this region (Figure 2A, GenBank accession numbers OQ974188 and OQ974189). There was a shorter transcript that stopped upstream of the miRNA cluster, and a longer transcript that terminated just downstream of the mir-463 gene. The longer transcript encoded 12 of the 19 miRNAs in the cluster, all of which were located in introns. In total, the full length lncRNA sequence encoding the mir-465 family in mouse liver was approximately 124 kb in length containing 14 exons, and was spliced into a 3746 bp non-coding transcript. The shorter lncRNA sequence was around 44 kb in length containing 12 exons, including a larger alternate exon 12, that stopped upstream of the miRNAs. The shorter transcript was spliced into a 3673 bp non-coding transcript. In order to determine if one, both, or none of these transcripts were transcribed and spliced in mouse liver, we performed PCR to identify the spliced products of each transcript using primers designed to the unique regions of each spliced transcript (Table 1; Figure 2B). We verified that both the long primary miRNA transcript encoding the miRNAs and the second, smaller transcript were produced and spliced in aged mouse liver and not in young mouse liver (Figure 2C), consistent with the expression pattern of the miRNAs.

### mir-465 family targets GHR in primary hepatocytes

**FIGURE 3:**
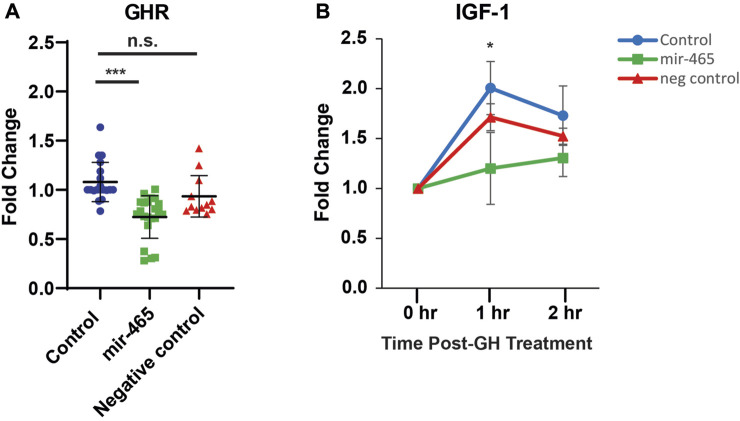
miR-465 attenuates GHR mRNA levels and GH signaling in culture primary hepatocytes. Primary hepatocytes were transfected with either a mir-465 mimic or a scrambled miRNA control mimic (negative control). When compared to non-transfected control primary hepatocytes (control), the mir-465 transfected primary hepatocytes showed a significant reduction in GHR mRNA levels **(A)**. Upon stimulation with growth hormone, mir-465 transfected hepatocytes showed a smaller increase in IGF-1 mRNA levels after 1 h when compared to non-transfected hepatocytes **(B)**. Error bars represent S.E.M.; *n* = 12–22. ***, *p* < 0.001; *, *p* < 0.05.

To determine if the reduction in GH signaling with age could be related to an increase in expression of the miRNA cluster, we validated that the mir-465 family targets the GHR in mouse liver hepatocytes. Previously, we established that all 3 members of the mir-465 family (mir-465a, mir-465b, and mir-465c) target the GHR 3′UTR equally in the immortalized AML12 mouse liver cell line, leading to a reduction in GHR at the mRNA and protein level (Elias et al., 2019). This resulted in an attenuation of JAK2/STAT5 signaling, and IGF-1 and IGFBP3 expression following GH stimulation (Elias et al., 2019). To verify that the mir-465 family targets the GHR leading to an attenuation of GH signaling in the liver we isolated primary hepatocytes from young mouse liver, since these cells are more physiologically relevant compared to the AML12 cell line used in our previous experiments, and transfected them with either a mir-465b mimic or a scrambled, control miRNA mimic. We investigated only 1 of the mir-465 family members since all 3 members act equally on the GHR mRNA (Elias et al., 2019). We found a significant 30% reduction in GHR mRNA expression in hepatocytes transfected with the mir-465 mimic when compared with non-transfected controls, whereas hepatocytes transfected with the control miRNA mimic did not show a significant reduction in GHR mRNA expression (Figure 3A). Forty-8 hours post-transfection the hepatocytes were exposed to 500 ng/mL of mouse growth hormone and followed over a 2-h period. Non-transfected hepatocytes and hepatocytes transfected with the control miRNA mimic had a significant increase in IGF-1 expression 1 h after GH stimulation. However, there was an attenuation of the increase in IGF-1 expression in response to GH stimulation in hepatocytes transfected with mir-465 (Figure 3B). These results showed that mir-465 family expression in primary hepatocytes led to a reduction in GHR expression and an attenuation of GH signaling.

### The mir-465 family targets the GHR *in vivo*

**FIGURE 4:**
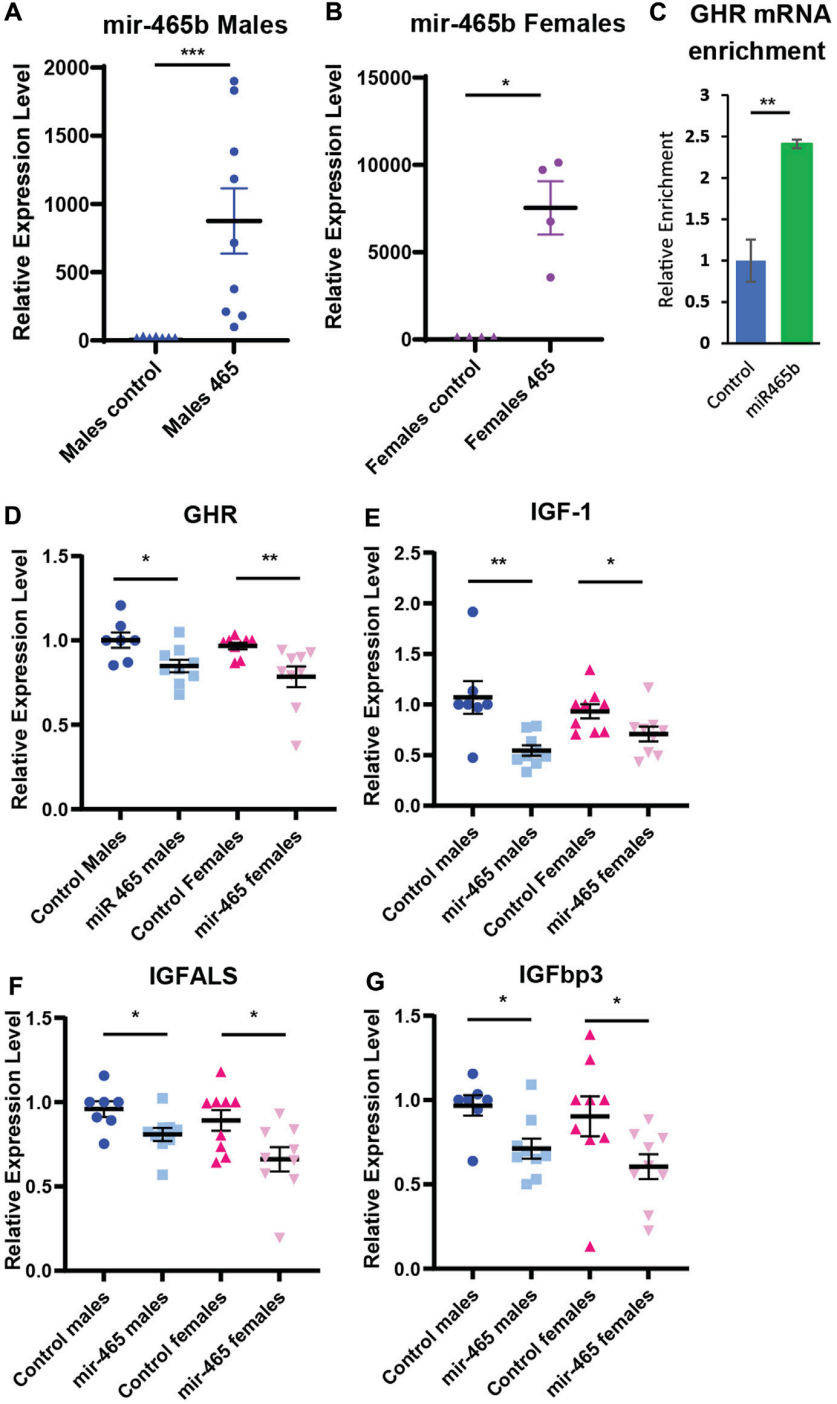
miR-465 attenuates GH signaling *in vivo.* Injection of a mir-465 mimic into the tail vein results in accumulation in the liver in **(A)** male and **(B)** female mice, shown as expression level normalized to snord66 and snord70. **(C)** GHR mRNA is enriched in a streptavidin pulldown of a biotinylated mir-465 mimic injected into the tail vein. Injection of a mir-465 mimic leads to a reduction in **(D)** GHR mRNA, **(E)** IGF-1 mRNA, **(F)** IGFALS mRNA, and **(G)** IGFBP3 mRNA in both male and female mouse liver after 4 days. All mRNA expression levels were normalized to GAPDH and HPRT. Error bars represent S.E.M.; *n* = 4–9. ***, *p* < 0.001; **, *p* < 0.01; *, *p* < 0.05.

Our *in vitro* data showed that the mir-465 family targets the GHR 3′UTR resulting in reduced GHR levels and an attenuation of GH signaling. To determine if the mir-465 family targets the GHR mRNA and leads to an attenuation of GH signaling *in vivo*, young male and female mice were injected with 2 nMole of a biotinylated mir-465b mimic, or a biotinylated scramble miRNA as a control, via the tail vein. Since we previously showed all 3 mir-465 family members target the GHR equally (Elias et al.), we only investigated one of the family members, mir-465b. At day 4 post injection, the liver was perfused through the portal vein with PBS to remove the blood and liver samples were taken for analysis. We found a significant increase in mir-465b in the liver in both male and female animals injected with the mir-465b mimic, indicating the miRNA was targeted to the liver (Figures 4A, B). There was no increase in mir-465b in animals injected with the scrambled miRNA control. A streptavidin pulldown of biotinylated miRNA mimics revealed an increase in GHR mRNA in the mir-465b mimic samples (Figure 4C), when compared to the scramble miRNA mimic control. These results suggested that mir-465b bound directly to the GHR mRNA in the mouse liver.

To verify that an increase in mir-465b levels results in decreased GH signaling, we examined several components of the GH signaling pathway. We found a significant reduction in GHR mRNA in both males and females (Figure 4D), indicating that mir-465b had similar activity in both sexes. GHR activation results in an increase in IGF-1 expression and in the associated proteins IGFBP3 and IGFALS. Therefore, a reduction in GHR expression should result in a reduction in expression of these factors as well. IGF-1 showed a 49% and 24% reduction in mRNA expression in males and females, respectively (Figure 4E). IGFBP3 showed a 27% and 34% reduction in mRNA levels in males and females, respectively (Figure 4F). We found that the IGFALS was also decreased in mRNA expression, by 17% and 26% in males and females, respectively (Figure 4G). These data indicated that an increase in levels of the mir-465 family in the liver *in vivo* led to a reduction in mRNA expression of the GHR, IGF-1, IGFBP3, and IGFALS, analogous to what is seen *in vitro*, and this pattern was similar in both males and females. The reduction in GHR and IGF-1 expression suggested a reduction in GH signaling, which has been shown to affect healthspan and lifespan.

**FIGURE 5:**
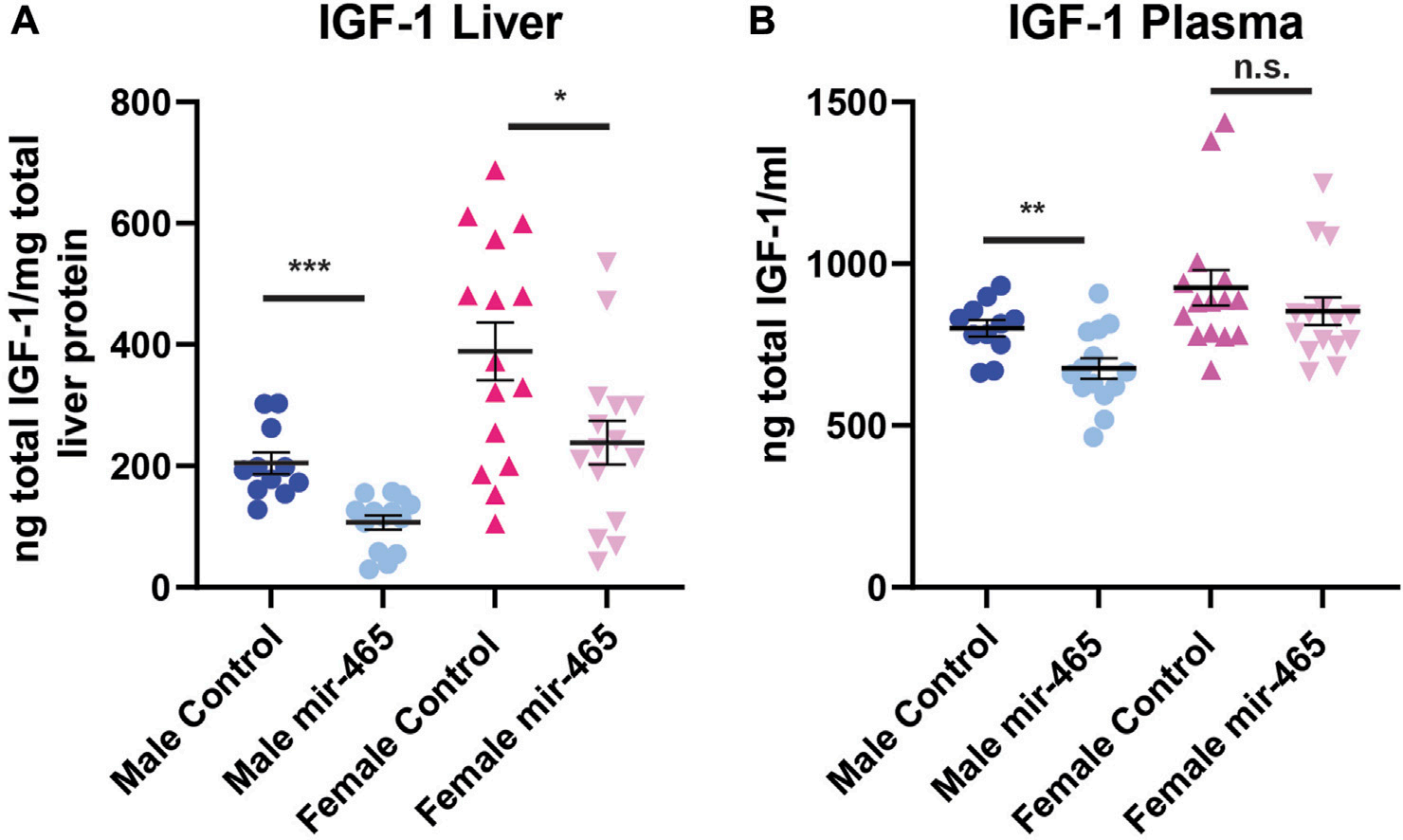
Liver and plasma IGF-1 protein levels following mir-465 injections. IGF-1 levels were measured in liver and plasma 4 days following injection of a mir-465 mimic or with a scrambled miRNA mimic control. **(A)** The liver of both males and females showed a significant reduction in IGF-1 protein following mir-465 injection. **(B)** IGF-1 protein levels were significantly reduced in the plasma in male mice, but there was no significant reduction in plasma levels of IGF-1 in females. Error bars represent S.E.M.; *n* = 11–14. ***, *p* < 0.001; **, *p* < 0.01; *, *p* < 0.05.

A reduction in mRNA levels of components of the GHR signaling pathway should translate to a reduction in the protein levels. To confirm this, we investigated total liver and plasma IGF-1 protein levels in the same mice. The majority of IGF-1 is normally bound by one of the IGF binding proteins (IGFBP) in a complex with ALS, preventing its detection by antibodies found in most ELISA kits. To circumvent this problem, the kit used here used low pH to dissociate IGF-1 from the IGFBP/ALS complex, and buffers with excess IGF-2 to out compete IGF-1 from re-binding to the IGFBPs. This allowed us to assay total IGF-1 levels, not just free IGF-1 levels. We found a 45% reduction in IGF-1 protein levels in the liver and a 15% reduction in the plasma of male mice injected with the mir-465b mimic, compared to mice injected with the scrambled sequence control miRNA mimic (Figures 5A, B). Female mice showed a 42% reduction in IGF-1 protein levels in the liver following mir-465b injection, however while there was a trend towards reduced plasma levels in females, the reduction was not significant (Figures 5A, B). These results indicated that there may be differential regulation of IGF-1 protein levels in the blood between males and females. We did note that overall, females had relatively higher IGF-1 levels in both the liver and the blood, and levels in females showed greater variation between individuals than in males.

## Discussion

We recently showed that a cluster of miRNAs located on the X-chromosome is de-repressed with age in mouse liver (Elias et al., 2019). Members of this cluster were previously shown to be expressed during spermatogenesis in the testes, in newborn ovary, and in pluripotent stem cells (Ahn et al., 2010; Zhao et al., 2014; Sherstyuk et al., 2017; Zhang et al., 2019a). In the testes aberrant expression of the cluster leads to defects in spermatogenesis suggesting that the miRNAs may regulate this process (Royo et al., 2015). Interestingly, they may also regulate stem cell differentiation, as members of the X-chromosomal cluster are upregulated during embryonic stem cell differentiation (Tay et al., 2008), and knocking out the cluster in rat fibroblasts inhibits reprogramming to induced pluripotent stem cells (Sherstyuk et al., 2019). The mir-465 family may also be involved in normal female placental development, as knocking out these miRNAs results in the sex ratio skewing towards males in mouse litters due to degeneration and resorption of female embryos (Wang et al., 2022). These studies indicate that the miRNAs in the X-chromosomal cluster can regulate developmental pathways. We and others have shown that this cluster is largely repressed in adult somatic tissues and is not expressed in liver in young mice (Elias et al., 2019; Ramaiah et al., 2019). However, we show that repression of this locus is lost in the liver of aged mice and there is a significant upregulation of expression of the miRNAs in this cluster. Our results show that despite being on the X-chromosome the expression levels are the same in males and females, suggesting that the inactivated X chromosome in females does not contribute to the miRNAs expression in females.

There is evidence that this cluster of miRNAs, located between *Slirtk2* and *Fmr1* on the X-chromosome in placental mammals, is rapidly evolving and evolutionarily conserved (Zhang et al., 2019a). In humans a 62 kb region between *Fmr1* and *Slitrk2* on the X-chromosome contains 21 miRNAs divided into 5 clusters that are primarily expressed in the testis (Keller et al., 2022). While the miRNA sequences are highly divergent between species, the predicted targets show >80% overlap between mouse and human, indicating that there is evolutionary pressure to retain their function. However, the mRNA targets of the X-cluster of miRNAs have not been well studied in mice or humans. Interestingly, several of the miRNAs in the cluster were shown to target the FMR1 gene, which is located adjacent to the X-miRNA cluster, and that the repression is additive (Ramaiah et al., 2019). In addition, 2 members of the cluster, mir-871-3p and mir-880-3p, target Fzd4, indicating that they can regulate Wnt signaling (Ota et al., 2019). The miRNAs in the cluster are predicted to impact PI3K-Akt signaling, mTOR signaling, MAPK signaling, Wnt signaling, and GH signaling in mice and all of these pathways are known to be dysregulated with age (Elias et al., 2019). Loss of repression of the miRNAs in the cluster could contribute to the age-related dysregulation of these pathways leading to the onset of age-associated changes. Previously, we showed that the mir-465 family targets the GHR mRNA and influences GH signaling *in vitro* (Elias et al., 2019). The data presented here shows that the GHR is targeted by this miRNA family *in vivo* in mouse liver leading to a reduction in GH signaling. These results have direct implications for aging, as GH and IGF-1 signaling are associated with modulating aging in a wide range of model systems.

Our data show that interventions to delay the onset of aging phenotypes also delay the de-repression of the X-chromosomal miRNA cluster. One of these interventions is calorie restriction. In *ad lib* fed mice the miRNA cluster is upregulated by 24 months of age. However, when animals were subjected to caloric restriction the increase in expression of the miRNA cluster was attenuated for most of the miRNAs that are located on the lncRNA encoding the mir-465 family (Figure 1C). While we did see an attenuation of the increase in expression of these miRNAs, there was a modest increase seen in calorie restricted animals consistent with the observation that calorie restriction induces a global upregulation of miRNAs (Zhang et al., 2019b). Interestingly, mir-883a shows a strong upregulation in calorie restricted animals when compared to *ad lib* fed animals (Figure 1C). This miRNA gene is located downstream of the lncRNA encoding the mir-465 family suggesting that miRNAs located in this region have different mechanisms of regulation than the mir-465 lncRNA. In addition, we see an attenuation of the increase in several of the miRNAs in 24-month-old Ames dwarf mice (Figure 1D). Of the miRNAs that are attenuated in this group all but one (mir-471) are located on the mir-465 lncRNA. Interestingly, mir-883a was not upregulated in the wild-type background strain of this mouse at 24 months, strengthening the hypothesis that miRNAs in this region are regulated differently than the mir-465 lncRNA.

A global reduction in GH signaling is associated with an increase in healthspan and lifespan, especially when the reduction occurs early in life. A global reduction in GHR expression led to improved insulin sensitivity, a reduction in cancer and an extension of lifespan in mice (Bellush et al., 2000; Ikeno et al., 2009; List et al., 2011; Haque et al., 2022). However, the timing of the GHR knockdown, and the sex of the animal, influenced the outcome. Globally knocking out GHR at sexual maturity at 1.5 months of age in mice led to improved insulin sensitivity, but reduced glucose tolerance in both sexes. Females had an extended maximal lifespan, where males did not (Junnila et al., 2016). Knocking down the GHR globally at 6 months of age had differential effects on males and females. Males had increased insulin sensitivity and reduced rates of cancer, but had no change in lifespan. Females had no change in insulin sensitivity or cancer rates, but showed a 15% increase in lifespan (Duran-Ortiz et al., 2021). Therefore, it appears that there are sex-specific effects of a global reduction in GH signaling, but overall a global reduction is beneficial for both sexes. Knocking out GH signaling in specific tissues has also yielded mixed results. Knocking out the GHR specifically in the liver throughout the lifespan led to insulin resistance and glucose intolerance in both sexes, and liver steatosis in males (Fan et al., 2009; List et al., 2014). However, a liver specific knockdown of GHR at 6 months was moderately protective against induced hepatocellular carcinoma (Haque et al., 2022). In addition, a liver specific reduction in IGF-1 in middle age led to liver inflammation, accelerated bone loss, increased hepatic tumors and an overall reduction in healthspan (Gong et al., 2014). We previously showed that the increase in mir-465 occurs around 24 months of age in mice, and this increase is most pronounced in liver (Elias et al., 2019). Given that the mir-465 family leads to a reduction in GH signaling later in life primarily in the liver, it is likely to have a detrimental effect on healthspan and lifespan.

Our results indicate that increased expression of the mir-465 family in liver, and the resulting reduction in GH signaling, leads to a reduction in circulating ternary complex consisting of IGF-1, IGFBP3 and ALS. We found a reduction in all 3 of these components in the liver and a reduction in circulating IGF-1 in the blood in mice injected with the mir-465 mimic**.** We saw greater inter-individual variation in IGF-1 levels in the liver and plasma of females in response to increased hepatic levels of mir-465 when compared to males (Figures 5A, B). This could be due to the female mice not being synchronized in their estrus cycle, since estrogen levels have been shown to influence circulating IGF-1 levels (Veldhuis et al., 2005; Ho et al., 2006). Reduced IGF-1 levels were shown to have both negative and positive consequences on aging phenotypes. A reduction in circulating IGF-1 levels was associated with a decline in cognitive function and sarcopenia in humans (Aleman et al., 1999; Aleman et al., 2000; Bian et al., 2020). However, in very old women (≥90 years) reduced circulating IGF-1 concentrations were associated with longer survival, where there was no association between circulating IGF-1 levels and survival in very old men (Milman et al., 2014). Along these same lines, female offspring of centenarians had higher levels of circulating IGF-1, whereas the same was not true for male offspring (Suh et al., 2008). In mice, reduced IGF-1 levels were protective against cardiomyocyte sensitivity to age related mechanical dysfunction (Li et al., 2008). These finding suggest that the reduction in circulating IGF-1 levels as a result of the increase in mir-465 expression could be both protective and deleterious, depending on the target tissue, and could contribute to the development of certain aging conditions.

Taken together, our results show that aging leads to dysregulation of a cluster of miRNAs on the X-chromosome. The mir-465 family directly targets the GHR mRNA *in vivo*, giving insight into potential mechanisms leading to a reduction in GH signaling with age. Interestingly, calorie restriction and the Ames dwarf genetic background, both known to delay the onset of aging phenotypes, do not show the same level of age-associated de-repression in mice. The targets of these miRNAs are evolutionarily conserved in placental mammals, including humans (Zhang et al., 2019a). Therefore, we expect to find that upregulation of these miRNAs produces similar results in humans.

## Data Availability

The datasets presented in this study can be found in online repositories. The names of the repository/repositories and accession number(s) can be found below: https://www.ncbi.nlm.nih.gov/genbank/, OQ974188, OQ974189.
